# Association of plasma free fatty acids levels with the presence and severity of coronary and carotid atherosclerotic plaque in patients with type 2 diabetes mellitus

**DOI:** 10.1186/s12902-020-00636-y

**Published:** 2020-10-21

**Authors:** Ming-Hua Zhang, Ye-Xuan Cao, Li-Guo Wu, Na Guo, Bing-Jie Hou, Li-Jing Sun, Yuan-Lin Guo, Na-Qiong Wu, Qian Dong, Jian-Jun Li

**Affiliations:** 1 Division of Dyslipidemia, State Key Laboratory of Cardiovascular Disease, Fu Wai Hospital, National Center for Cardiovascular Diseases, Chinese Academy of Medical Sciences and Peking Union Medical College, No 167 BeiLiShi Road, XiCheng District, Beijing, 100037 China; 2 Department of Internal Medicine, The People’s Hospital of Tang Xian County, Baoding, 072350 Hebei China

**Keywords:** Free fatty acids, Coronary artery disease, Carotid atherosclerotic plaque, Diabetes mellitus

## Abstract

**Background:**

Previous studies have suggested that patients with diabetes mellitus (DM) have higher prevalence of atherosclerotic cardiovascular disease (ASCVD), and plasma levels of free fatty acids (FFAs) are a useful marker for predicting ASCVD. We hypothesized that FFAs could predict both coronary and carotid lesions in an individual with type 2 DM (T2DM). The present study, hence, was to investigate the relation of plasma FFA level to the presence and severity of coronary and carotid atherosclerosis in patients with T2DM.

**Methods:**

Three hundred and two consecutive individuals with T2DM who have received carotid ultrasonography and coronary angiography due to chest pain were enrolled in this study. Plasma FFAs were measured using an automatic biochemistry analyzer. Coronary and carotid severity was evaluated by Gensini score and Crouse score respectively. Subsequently, the relation of FFA levels to the presence and severity of coronary artery disease (CAD) and carotid atherosclerotic plaque (CAP) in whole individuals were also assessed.

**Results:**

Increased plasma FFA levels were found in the groups either CAD or CAP compared to those without. Patients with higher level of FFAs had a higher CAD (89.9%) and elevated prevalence of CAP (69.7%). And also, patients with higher level of FFAs had a higher Gensini and Crouse scores. Multivariate regression analysis showed that FFA levels were independently associated with the presence of CAD and CAP (OR = 1.83, 95%CI: 1.27–2.65, *P* = 0.001; OR = 1.62, 95%CI: 1.22–2.14, P = 0.001, respectively). The area under the curve (AUC) was 0.68 and 0.65 for predicting the presence of CAD and CAP in patients with DM respectively.

**Conclusions:**

The present study firstly indicated that elevated FFA levels appeared associated with both the presence and severity of CAD and CAP in patients with T2DM, suggesting that plasma FFA levels may be a useful biomarker for improving management of patients with T2DM.

## Background

Atherosclerosis (AS) is a chronic systemic disease, which is involved in multiple, large and medium-sized arteries, resulting in the different clinical phenotypes including coronary artery disease (CAD), strokes and peripheral vascular disease (PVD). Moreover, AS has also been considered as multiple cause disorder, which is associated with dyslipidemia, hypertension, smoking, overweight, and diabetes mellitus (DM) [1]. Among them, DM is named as equivalent risk fact as CAD due to its higher multiple vessel lesions in a individual.

As is well known, DM has become a major health and economic problem in both developing and developed countries, especially in China [1]. Recent data has suggested that there are one hundred and thirty million individuals with DM. More importantly, pre-diabetes mellitus (Pre-DM), an intermediate state between normal glucose regulation (NGR), is as high as 35.7% morbidity rate in China [2]. Thereby, much attention should be paid regarding the relation of risk factor to the development of DM [2].

In fact, it has been previously demonstrated that DM is a strong, independent risk factor for AS [3]. Their clinical phenotypes may clinically presented as CAD or carotid plaque. Besides, previous studies have shown that at least 30% of ischemic strokes occur in the carotid distribution area, which is a consequence of severe carotid stenosis caused by atherosclerotic occlusive disease [4, 5]. Thereby, exploration the potential common biomarker for predicting coronary and carotid atherosclerotic plaque (CAP) would be greatly interesting clinically.

Free fatty acid (FFA), named as non-esterified fatty acid also, is a product of lipid metabolism and one of the main energy-supplying substances in the human body. It provides energy for the metabolism of heart, liver, skeletal muscle and other organs. Interestingly, previous studies including ours have indicated that elevated plasma FFA levels were related to the incidence and prognosis of CAD, including increased risk of arrhythmia, acute myocardial infarction, sudden cardiac death and all-cause mortality [6–9]. Moreover, only two previous studies with small sample size suggested that FFA was the risk factor for carotid atherosclerosis in patients with DM [10, 11]. In this study, we investigated the potential role of plasma FFA level in predicting the presence and severity of coronary and carotid atherosclerosis in patients with type 2 DM (T2DM).

## Methods

### Patients

From December 2016 to November 2018, 1026 consecutive patients with T2DM were screened out from Dyslipidemia and Cardiovascular Disease center in FuWai Hospital. The inclusion criteria for patients with T2DM were as follows: (1) men and women over 18 years old; (2) underwent coronary angiography and carotid ultrasonography. Exclusion criteria included: (1) patients whose data is not entire; (2) severe liver or/and renal insufficiency; (3) thyroid dysfunction; (4) malignant disease; (5) familial hypercholesterolemia (FH). As a result, a total of 302 eligible patients were recruited in the present study.

The study protocol was in compliance with the Declaration of Helsinki, and was approved by the hospital’s ethics review committee (Fu Wai Hospital and the National Center for Cardiovascular Diseases, Beijing, China, approval number: 2013–442). Informed written consents were obtained from all patients enrolled in this study.

### Clinical data collection

The clinical characteristics after admission were collected from all enrolled patients including age, gender, body mass index (BMI), the medical history of hypertension (HTN), stroke, smoking status and the usage of aspirin, statins, angiotensin converting enzyme inhibitors (ACEI), angiotensin receptor blocker (ARB) and Calcium Blocker. DM was defined by fasting plasma glucose (FPG) ≥7.0 mmol/L or the 2-h plasma glucose of the oral glucose tolerance test ≥11.1 mmol/L or currently using hypoglycemic drugs or insulin. BMI was calculated as BMI (kg/m^2^) = body weight (kg)/body height (m^2^). HTN was diagnosed as a self-reported hypertension, systolic blood pressure (SBP) ≥140 mmHg and/or diastolic blood pressure (DBP) ≥90 mmHg for three or more consecutive times or currently taking antihypertensive drugs. Smoking was ascertained as participants who had smoked regularly within the previous 12 months. CAD was defined as coronary stenosis more than 50% of at least one coronary artery.

### Laboratory analysis

Blood samples of all the enrolled patients were collected from cubital vein after a 12 h overnight fasting and collected into EDTA-containing tubes. Plasma concentrations of total cholesterol (TC), triglyceride (TG), high-density lipoprotein cholesterol (HDL-C), low-density lipoprotein cholesterol (LDL-C), apolipoprotein A (apoA), apolipoprotein B (apoB), FPG and FFAs were measured using an automatic biochemistry analyzer (Hitachi 7150, Tokyo, Japan) as our previous published papers [12, 13]. Plasma hemoglobin A1C (HbA1c) levels were measured using the Tosoh G7 Automate HPLC Analyzer (TOSOH Bioscience, Japan).

### Coronary angiography and the coronary severity evaluation

All enrolled patients in the present study underwent coronary angiography using standard Judkins techniques according to our previous studies [12, 14]. CAD was mainly manifested as coronary artery diameter stenosis of more than 50%. The severity of coronary atherosclerosis was evaluated by the Gensini score, which assigned the severity score based on the degree and location of stenosis based on our previous publications [15]. In detail, the narrowing was scored as 32 for 100% occluded artery, 16 for 91–99%, 8 for 76–90%, 4 for 51–75%, 2 for 26–50%, and 1 for 1–25%. The score was then multiplied by a coefficient to indicate the functional importance of each segment. The coefficient was as follows: the left main coronary artery was 5, the proximal left anterior descending artery and the proximal circumflex artery were 2.5, the middle left anterior descending artery was 1.5, the right coronary artery, the distal left anterior descending artery, the posterolateral artery and obtuse artery were 1.0, and the residual major segments were 0.5 [16]. Finally, the Gensini score was calculated by the sum of the integral of each coronary artery segment [16].

### Carotid ultrasound examination

Each patient’s bilateral carotid arteries were examined by two experienced ultrasound physicians who were blind to the clinical characteristics of the patients using 128 System (Acuson, Mountain View, CA, USA) with a high-resolution 7.5–10.0 MHz transducer accrdoing to our previous study [12, 17]. The intima-media thickness (IMT) of proximal and distal common carotid artery, bifurcation of left and right common carotid artery and carotid sinus were measured respectively. Carotid plaque was defined as the IMT exceeding 1.5 mm, or local IMT enlargement exceeding 50% of surrounding IMT, and recorded as presence or absence. Crouse score for carotid plaques was defined as the sum of maximum thicknesses of all plaques as previously reported [18].

### Statistical analysis

The values were expressed as the mean ± standard deviation (SD) or median (Q1-Q3 quartiles) for the continuous variables and the number (percentage) for the categorical variables. The differences of parameters among groups were compared by students’ t-test, variance analysis (ANOVA), Mann-Whitney U test or Chi-square test as appropriate. Spearman’s correlation coefficients were used to assess the correlations between parameters. Univariable and multivariable logistic regression analysis was performed to determine the odds ratios (ORs) and 95% confidential intervals (CIs) of FFAs and CAD and CAP, adjusted by potential confounding factors.The diagnostic value of FFAs on the presence of CAD and CAP was analyzed by receiver operating characteristic curves (ROC). A *p* value < 0.05 was defined as statistically significant. The statistical analysis was performed using SPSS version 25.0 software (SPSS Inc., Chicago Illinois, USA).

## Results

### Patient characteristics

We divided 302 patients into CAD group and without CAD group and CAP group and without CAP group. The baseline characteristics and laboratory data of all patients were shown in Table 1. As is shown in the Table 1, the average age of the studied patients was 57.52 ± 10.56 years, of which 64.9% were males. The mean plasma FFA level was 0.49 ± 0.17 mmol/l.

**Table 1:** Baseline characteristics in patients with or without CAD and CAP

Variables	Total	CAD	Non-CAD	P value	CAP	Non-CAP	P value
	(***n*** = 302)	(***n*** = 232)	(***n*** = 70)		(***n*** = 165)	(***n*** = 137)	
***Baseline characteristics***							
Age, years	57.52 ± 10.56	58.08 ± 10.48	55.66 ± 10.72	0.093	59.10 ± 11.01	55.61 ± 9.70	**0.004**
Male, n (%)	196 (64.9)	163 (70.3)	33 (47.1)	**< 0.001**	113 (68.5)	83 (60.6)	0.152
BMI, kg/m^2^	26.43 ± 3.15	26.36 ± 3.07	26.57 ± 3.41	0.648	26.32 ± 3.11	26.51 ± 3.20	0.606
Family history of CAD, n (%)	42(13.9)	32(13.8)	10(14.3)	0.855	22(13.3)	20(14.6)	0.758
HTN, n (%)	217 (71.9)	177 (76.3)	40 (57.1)	**0.002**	127 (77.0)	90 (65.7)	**0.030**
Stroke, n (%)	17 (5.6)	12 (5.2)	5(7.1)	0.519	12 (7.3)	5 (3.6)	0.192
Smoking, n (%)	154 (51.0)	126(54.3)	28 (40.0)	**0.028**	91 (55.2)	63 (46.0)	0.129
Alcohol drinker, n(%)	102 (33.8)	80(34.5)	22 (31.4)	0.557	63 (38.2)	39 (28.5)	0.085
***Laboratory parameters***							
FPG, mmol/L	7.42 ± 2.16	7.63 ± 2.27	6.68 ± 1.54	**< 0.001**	7.57 ± 2.20	7.24 ± 2.11	0.185
HbA1c (%)	7.33 ± 1.32	7.49 ± 1.36	6.77 ± 0.98	**< 0.001**	7.41 ± 1.29	7.24 ± 1.35	0.289
TC, mmol/L	4.29 ± 1.29	4.21 ± 1.32	4.55 ± 1.16	0.056	4.39 ± 1.45	4.17 ± 1.06	0.127
LDL-C, mmol/L	2.61 ± 1.10	2.55 ± 1.13	2.83 ± 0.99	0.066	2.70 ± 1.26	2.51 ± 0.86	0.114
HDL-C, mmol/L	1.02 ± 0.28	1.01 ± 0.29	1.03 ± 0.26	0.697	1.03 ± 0.29	1.00 ± 0.26	0.315
TG, mmol/L	1.64(1.25–2.41)	1.62(1.22–2.39)	1.93(1.31–2.63)	0.211	1.69(1.21–2.58)	1.58(1.29–2.37)	0.854
ApoA, g/L	1.33 ± 0.43	1.33 ± 0.47	1.37 ± 0.26	0.481	1.37 ± 0.52	1.29 ± 0.28	0.131
ApoB, g/L	0.90 ± 0.30	0.88 ± 0.30	0.94 ± 0.29	0.168	0.89 ± 0.32	0.91 ± 0.27	0.52
Lp(a), mg/dL	115.34 (53.68–281.85)	112.35 (53.9–299.98)	122.1 (52.95–231.38)	0.933	110.1 (60.25–253.0)	125.7 (45.1–334.6)	0.622
Hs-CRP, mg/dL	1.45(0.77–2.78)	1.46(0.77–2.73)	1.39(0.69–2.87)	0.824	1.51(0.87–2.87)	1.37 (0.61–2.65)	0.289
LVEF (%)	63.23 ± 7.44	62.67 ± 7.92	65.04 ± 5.23	**0.021**	62.64 ± 7.69	63.91 ± 7.10	0.147
FFAs, mmol/L	0.49 ± 0.17	0.51 ± 0.17	0.41 ± 0.15	**< 0.001**	0.54 ± 0.17	0.44 ± 0.15	**< 0.001**
***Medicines***							
Aspirin, n (%)	177 (58.6)	154 (66.4)	23 (32.9)	**0.002**	92 (55.8)	85 (62.0)	0.147
Statin, n (%)	145 (48.0)	122 (52.6)	23 (32.9)	**0.004**	81 (49.1)	64 (46.7)	0.681
ACEI, n (%)	34 (11.3)	32 (13.8)	2 (2.9)	0.071	20 (12.1)	14 (10.2)	0.524
ARB, n (%)	44 (14.6)	36 (15.5)	8 (11.4)	0.652	24 (14.5)	20 (14.6)	0.824
Calcium Blocker, n (%)	60 (19.9)	48(20.7)	12 (17.1)	0.390	31 (18.8)	29 (21.2)	0.764

Data are expressed as mean ± SD, median (25th–75th percentile) or n (%).Bold values indicate statistical significance. *CAD* coronary artery disease, *CAP* carotid atherosclerotic plaque, *BMI* body mass index, *HTN* hypertension, *FPG* fasting plasma glucose, *HbA1C* hemoglobin A1C, *TC* total cholesterol, *LDL-C* LDL cholesterol, *HDL-C* HDL cholesterol, *TG* triglyceride, *Lp(a)* lipoprotein(a), *Hs-CRP* high sensitivity C-reactive protein, *LVEF* left ventricular ejection fraction, *FFAs* free fatty acids, *ACEI* angiotensin converting enzyme inhibitors, *ARB* angiotensin receptor blocker

As is shown in Table 2, the 302 patients were divided into three groups according to the tertiles of plasma level of FFAs: tertile 1 (< 0.41 mmol/L, *n* = 107), tertile 2 (0.41–0.56 mmol/L, *n* = 96), tertile 3 (> 0.56 mmol/L, *n* = 99). Patients with the higher levels of FFAs appeared to have increased concentrations of FPG and HbA1C when compared with the first tertile (all *p* value for trend < 0.05).

**Table 2:** Demographic characteristics stratified by FFAs tertiles (mmol/L)

Variables	Tertile 1	Tertile 2	Tertile 3	***P***-value
	(< 0.41)	(0.41–0.56)	(> 0.56)	
	N = 107	N = 96	N = 99	
***Baseline characteristics***				
Age, years	57.19 ± 9.27	55.81 ± 12.01	59.53 ± 10.17	0.045
Male, n (%)	65(60.7)	67(69.8)	64(64.6)	0.402
BMI, kg/m^2^	26.09 ± 3.03	27.00 ± 3.42	26.20 ± 2.95	0.092
Family history of CAD, n (%)	14(13.1)	18(18.8)	10(10.1)	0.173
HTN, n (%)	76(71.0)	66(68.8)	75(75.8)	0.538
CAD, n (%)	71(66.4)	72(75.0)	89(89.9)	**< 0.001**
Stroke, n (%)	6(5.6)	5(5.2)	6(6.1)	0.972
Smoking, n (%)	53(49.5)	54(56.3)	47(47.5)	0.516
Alcohol drinker, n (%)	36(33.6)	33(34.4)	33(33.3)	0.993
Carotid plaque, n (%)	43(40.2)	53(55.2)	69(69.7)	**< 0.001**
***Laboratory parameters***				
FPG, mmol/L	6.89 ± 1.96	7.57 ± 2.01	7.84 ± 2.40	**0.005**
HbA1c (%)	7.15 ± 1.21	7.21 ± 1.34	7.63 ± 1.36	**0.022**
TC, mmol/L	4.20 ± 1.19	4.13 ± 1.31	4.54 ± 1.35	0.060
LDL-C, mmol/L	2.55 ± 0.97	2.56 ± 1.13	2.72 ± 1.20	0.469
HDL-C, mmol/L	1.00 ± 0.28	0.99 ± 0.27	1.07 ± 0.29	0.097
TG, mmol/L	1.46(1.27–2.21)	1.63(1.25–2.36)	1.82(1.20–2.88)	0.127
ApoA, g/L	1.29 ± 0.24	1.35 ± 0.67	1.38 ± 0.25	0.295
ApoB, g/L	0.90 ± 0.30	0.88 ± 0.30	0.91 ± 0.29	0.697
Lp(a), mg/dL	116.75(51.55–354.49)	156.4(60.33–316.70)	95.3(52.70–214.20)	0.121
Hs-CRP, mg/dL	1.28(0.63–2.82)	1.47(0.84–2.98)	1.58(0.82–2.50)	0.509
FFAs, mmol/L	0.32 ± 0.07	0.49 ± 0.04	0.68 ± 0.11	**<0.001**
Gensini score	19.0(8.0–41.5)	30.0(8.0–64.0)	46.0(16.0–86.0)	**<0.001**
Crouse score	2.40 ± 1.47	3.02 ± 1.55	3.34 ± 1.73	**<0.001**
***Medicines***				
Aspirin, n (%)	56(52.3)	56(58.3)	65(65.7)	0.496
Statin, n (%)	48(44.9)	48(50.0)	49(49.5)	0.717
ACEI, n (%)	11(10.3)	15(15.6)	8(8.1)	0.278
ARB, n (%)	17(15.9)	15(15.6)	12(12.1)	0.519
Calcium Blocker, n (%)	21(19.6)	19(19.8)	20(20.2)	0.956

Data are expressed as mean ± SD, median (25th–75th percentile) or n (%). Bold values indicate statistical significance. *BMI* body mass index, *HTN* hypertension, *CAD* coronary artery disease, *FPG* fasting plasma glucose, *HbA1C* hemoglobin A1C, *TC* total cholesterol, *LDL-C* LDL cholesterol, *HDL-C* HDL cholesterol, *TG* triglyceride, *Lp(a)* lipoprotein(a), *Hs-CRP* high sensitivity C-reactive protein, *FFAs* free fatty acid, *ACEI* angiotensin converting enzyme inhibitors, *ARB* angiotensin receptor blocker

### Relation of FFAs to CAD

**Fig. 1:**
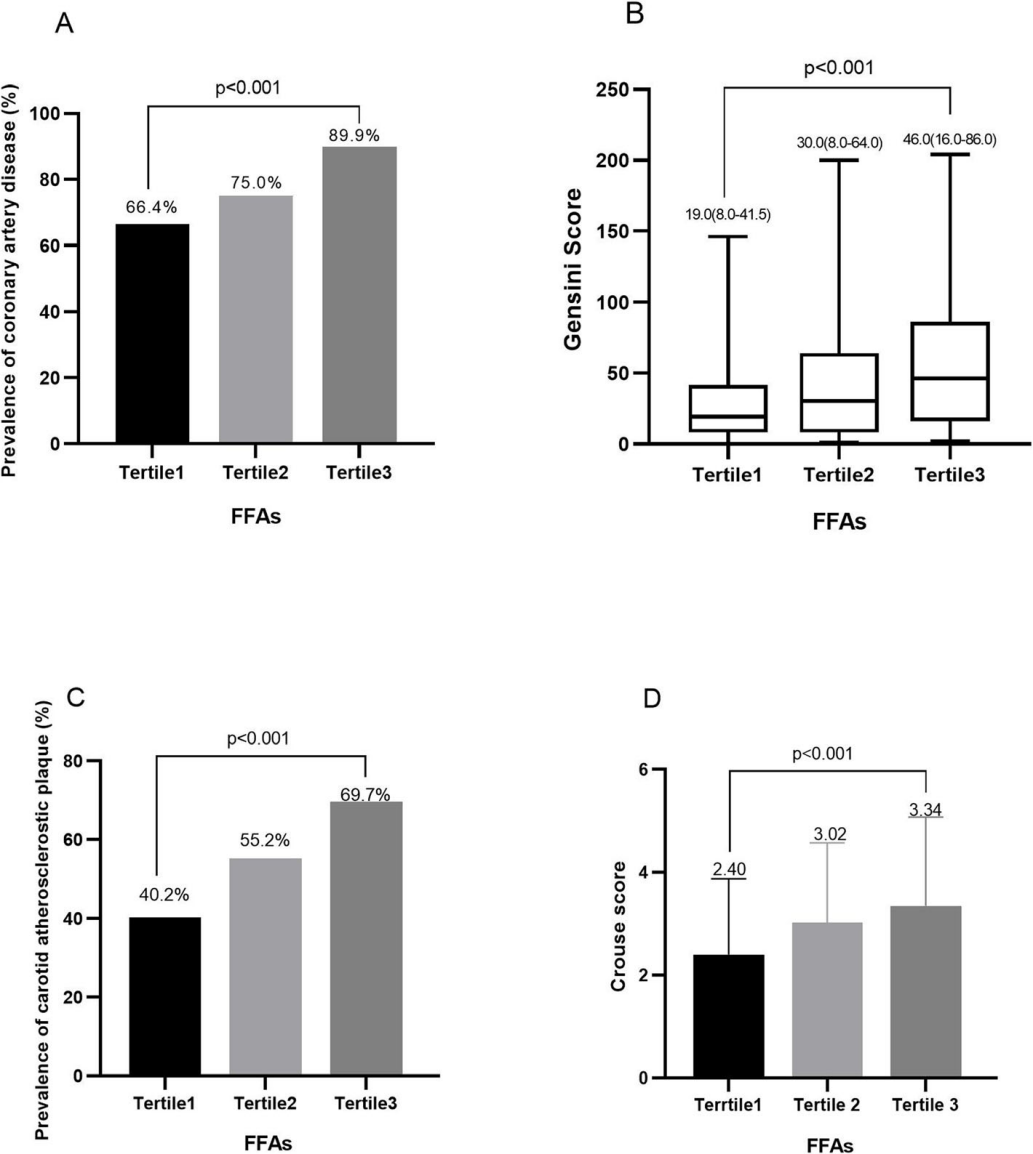
Association between free fatty acids and the presence and severity of coronary artery disease and carotid atherosclerotic plaque

As is shown in Table 1, CAD was present in 232 patients (76.8%) and there were more often men (70.3%) with a higher history of HTN (76.3%) and smoking (54.3%). Importantly, plasma FFA levels were significantly elevated in patients with CAD compared with that without (0.51 ± 0.17 mmol/l vs 0.41 ± 0.15 mmol/L, *p* < 0.001). There were lower left ventricular ejection fraction (LVEF) and higher FPG, HbA1c and rates of aspirin and statins in patients with CAD (all p for trend < 0.05). Besides, comparing the patients with low, medium and high FFA levels, we found significant differences in the prevalence of CAD and Gensini scores (*p* < 0.001 in all, Table 2, Fig. 1a and b).

In addition, the results showed that FFAs were positively associated with CAD presence (r = 0.257, p < 0.001) and Gensini scores (r = 0.268, p < 0.001) in the Spearman correlation analysis (Table 3). Moreover, statin therapy, FPG and HbA1c were positively associated with the presence of CAD and Gensini scores. In the multivariable model (Table 4), FFA per SD increase was associated with increased risk of the presence of CAD (OR = 1.83, 95%CI: 1.27–2.65, *p* = 0.001) after adjusting traditional risk factors including age, gender, BMI, statin therapy, HTN, smoking, FPG, HbA1C, TC, HDL-C and LDL-C. Besides, gender, HTN, statin therapy and HbA1c were also predictors for the presence of CAD.

**Table 3:** Correlation between risk factors and the presence and severity of coronary artery disease and carotid atherosclerotic plaque

Variables	CAD		Gensini score		Carotid plaque		Crouse score	
	r	p-value	r	p-value	r	p-value	r	p-value
Age,year	0.113	0.050	0.112	0.067	0.149	**0.009**	0.144	**0.012**
Male, n(%)	−0.204	**< 0.001**	− 0.068	0.265	− 0.082	0.153	− 0.090	0.118
BMI, kg/m 2	−0.017	0.780	−0.046	0.461	−0.016	0.784	0.007	0.906
Smoking, n(%)	−0.127	**0.028**	−0.045	0.469	−0.088	0.130	−0.085	0.144
Statin, n(%)	−0.167	**0.004**	−0.133	**0.029**	−0.024	0.682	−0.014	0.802
FPG, mmol/L	0.184	**0.001**	0.129	**0.035**	0.079	0.173	0.117	**0.044**
HbA1c (%)	0.247	**< 0.001**	0.189	**0.002**	0.073	0.211	0.049	0.399
TC, mmol/L	−0.111	0.056	−0.100	0.102	0.060	0.297	0.024	0.675
LDL-C,mmol/L	−0.106	0.066	−0.087	0.157	0.048	0.412	0.060	0.300
Lpa, mg/dl	0.005	0.933	0.049	0.421	−0.029	0.622	−0.020	0.727
Hs-CRP,mg/l	0.013	0.825	0.060	0.327	0.061	0.290	0.106	0.066
FFAs, mmol/L	0.257	**< 0.001**	0.268	**< 0.001**	0.254	**< 0.001**	0.359	**< 0.001**

Bold values indicate statistical significance. *BMI* body mass index, *FPG* fasting plasma glucose, *HbA1C* hemoglobin A1C, *TC* total cholesterol, *LDL- C* LDL cholesterol, *Lp(a)* lipoprotein(a), *Hs-CRP* high sensitivity C-reactive protein, *FFAs* free fatty acids

**Table 4:** Univariate and multivariate analysis for the association between FFAs and the presence of coronary artery disease and carotid atherosclerotic plaque

Variables	Univariate ^**a**^		Multivariate ^**a**^		Univariate ^**b**^		Multivariate ^**b**^	
	OR (95%CI)	P-value	OR (95%CI)	P-value	OR (95%CI)	P-value	OR (95%CI)	P-value
Age	1.03(1.00–1.07)	0.129	1.04(1.00–1.07)	0.063	1.05(1.02–1.08)	0.003	1.04(1.02–1.07)	**0.003**
Gender	3.78(1.51–9.46)	0.004	3.91(1.89–8.01)	**< 0.001**	1.79(0.87–3.68)	0.114	1.87(1.06–3.31)	**0.032**
HTN	2.31(1.17–4.62)	0.016	2.12(1.01–4.13)	**0.027**	1.71(0.96–3.03)	0.068	1.67(0.96–2.91)	0.072
Smoking	0.84(0.37–1.86)	0.66			1.22(0.64–2.31)	0.545		
Statin	1.92(0.95–3.88)	0.069	2.12(1.11–4.07)	**0.024**	0.92(0.54–1.59)	0.772		
BMI	0.94(0.85–1.04)	0.237			0.99(0.91–1.08)	0.740		
FPG	1.12(0.92–1.38)	0.266			1.01(0.88–1.16)	0.859		
HbA1C	1.55(1.01–2.17)	0.013	1.70(1.25–2.30)	**0.001**	1.04(0.83–1.30)	0.729		
TC	0.89(0.49–1.61)	0.705			1.04(0.65–1.65)	0.879		
HDL-C	0.78(0.22–2.72)	0.691			0.97(0.35–2.74)	0.958		
LDL-C	0.96(0.50–1.85)	0.908			1.14(0.68–1.92)	0.611		
FFAs per SD	1.89(1.29–2.77)	0.001	1.83(1.27–2.65)	**0.001**	1.60(1.20–2.14)	0.002	1.62(1.22–2.14)	**0.001**

^a^ coronary artery disease; ^b^ carotid atherosclerotic plaque; *OR* odds ratio, *CI* confidence interval, *HTN* hypertension, *BMI* body mass index, *FPG* fasting plasma glucose, *HbA1C* hemoglobin A1C, *TC* total cholesterol, *HDL-C* HDL cholesterol, *LDL- C* LDL cholesterol, *FFAs* free fatty acids

### Association of FFAs with CAP

The data from Table 1 indicated that patients with CAP were older (59.10 ± 11.01 vs 55.61 ± 9.70, *p* = 0.004), more often male (68.5%) and had higher rate of HTN (77.0%). Plasma FFA levels were significantly elevated in patients with CAP compared with that without (0.54 ± 0.17 mmol/l vs 0.44 ± 0.15 mmol/L, *p* < 0.001). We also compared the patients with low, medium and high FFA levels and found significant differences in the prevalence of CAP and Crouse scores (all *p* value < 0.001; Table 2, Fig. 1c and d).

Moreover, spearman correlation analysis (Table 3) showed that FFAs were positively correlated with the presence of CAP (r = 0.254, p < 0.001) and Crouse scores (r = 0.359, p < 0.001). Additionally, data suggested that age and FPG were positively correlated with Crouse scores. After adjusting traditional risk factors including age, gender, BMI, statin therapy, HTN, smoking, FPG, HbA1C, TC, HDL-C and LDL-C, FFA per SD increase was associated with increased risk of the presence of CAP in multivariable regression analysis (OR = 1.62, 95%CI: 1.22–2.14, *p* = 0.001, Table 4). Besides, age and gender were also predictors for the presence of CAP.

### Diagnostic value of FFAs on CAD and CAP

**Fig. 2:**
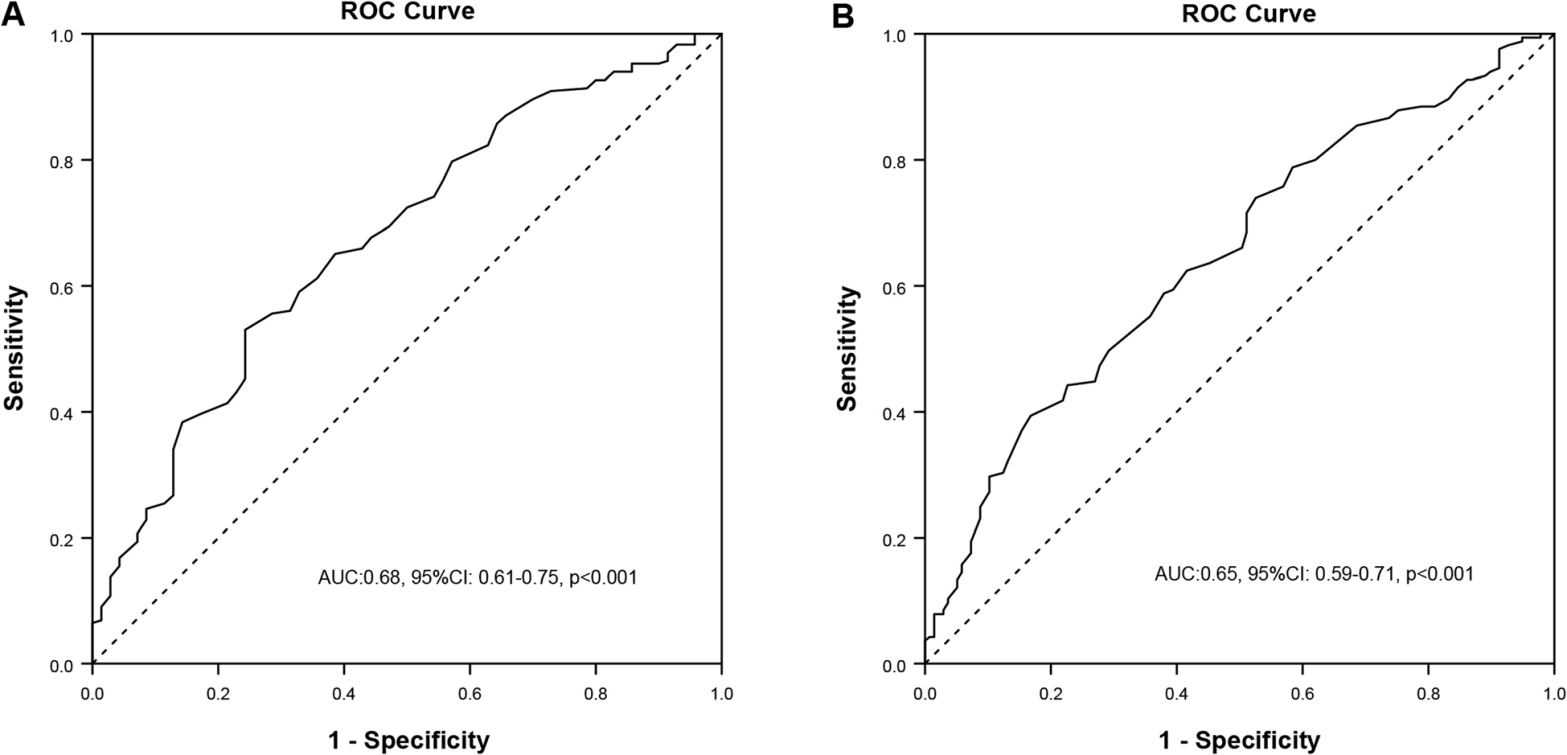
The area under the curve of free fatty acids in predicting the presence of coronary artery disease (**a**) and carotid atherosclerotic plaque (**b**). AUC: area under the curve; ROC: receiver operating characteristics.

As is shown in Fig. 2 a and b, the area under the curve (AUC) indicated that the FFAs had an appropriate discriminate ability in diagnosing the presence of CAD (AUC = 0.68, 95% CI: 0.61–0.75, *p* < 0.001) and CAP (AUC = 0.65, 95% CI: 0.59–0.71, p < 0.001) in patients with T2DM.

## Discussion

Compared with what we have known about the role of FFAs in the presence, severity, and clinical outcomes in patients with CAD, less is known in the predicting value of FFAs in different sites of AS. In another word, the value of FFAs for predicting atherosclerotic cardiovascular disease (ASCVD) in T2DM has been less investigated, especially in the presence and severity of CAD and CAP. In the present study, we consecutively enrolled 302 patients with T2DM who underwent coronary angiography and carotid ultrasonography for the purpose of examining the relationship between FFAs and the presence and severity of CAD and CAP. The major novel findings were as follows: 1) the patients with CAD and CAP had higher FFA levels; 2) plasma FFA level was independently associated with the presence of both CAD and CAP after adjustment for traditional risk factors. These finding might improve our understandings regarding the association of FFAs with AS.

Although FFAs have been found for many years, it still have important significance for clinical diagnosis and prognosis of metabolic diseases. According to previous studies, FFAs play an important role in cell membrane formation, cell signal transduction and regulation of glucose metabolism [19, 20]. Moreover, recent accumulated evidence has confirmed that FFAs were involved in the pathogenesis of insulin resistance and subsequent development of metabolic syndrome such as obesity and T2DM [21]. Subsequent studies further suggested that excess of plasma FFAs can induce oxidative stress, inflammation and vascular endothelial cell damage [22–24], which leads to accelerated rupture of atherosclerotic plaque and disease progression. In addition, elevated plasma FFAs also contributed to HDL dysfunction based on the fact that they increased the concentration of TG in HDL particles, which is considered to be a factor affecting AS [25].

In fact, previous studies have also indicated that FFAs is a useful marker for predicting the presence and severity of several cardiovascular diseases, particularly in CAD. Stefan [7] et al. have proved the independent association of FFAs with all-cause and cardiovascular mortality in 2567 patients with CAD for 5.38 years follow-up. Olga [26] et al. have enrolled 79 patients with ST elevation myocardial infarction and found that FFAs were associated with a higher risk of myocardial infarction and acute heart failure. Moreover, Taniguchi et al. [10, 11] have revealed the association of serum non-esterified fatty acids with carotid atherosclerosis (IMT in plaque-free segments and carotid stenosis in plaque segments) in 54 non-obese, non-hypertensive, well-controlled Japanese patients with T2DM. However, there is no data available regarding the relationship between plasma FFAs and the presence and severity of CAD and CAP in an individual with T2DM. That is the reason why we performed the present study.

Interestingly, we found that the patients with CAD and CAP had nearly more that 20% increase in plasma FFA levels (CAD:0.51 ± 0.17 mmol/L vs 0.41 ± 0.15 mmol/L, CAP: 0.54 ± 0.17 mmol/L vs 0.44 ± 0.15 mmol/L, respectively). In addition, we used Gensini and Crouse scores to examine the relation of FFAs to the severity of CAD and CAP. We found that the significantly correlations between Gensini and Crouse scores and FFA levels and patients with higher plasma FFA levels had higher Gensini and Crouse scores. Finally, this study confirmed that FFAs were independently associated with the presence of CAD and CAP in patients with DM after adjusting for confounding risk factors.

The clinical implication of the present study was that the FFAs might be involved in not only CAD but also CAP in patients with T2DM. Therefore, the data in this study provided additional clues for developing more effective secondary prevention and treatment strategies for the patients with T2DM. Furthermore, in view of fact that the measurement of plasma FFA levels is convenient and cost-effective, it is better to be commonly measured in diabetic patients with CAD or high risk for cardiovascular disease, which is helpful to disease stratification and treatment in clinical practice.

Although the present provided additional information concerning the relationship between FFAs and multiple site of AS, there were several limitations in our study. Firstly, this is a single center study with small sample size, which may lead to potential selection bias. Moreover, because the concentration of FFAs in blood is affected by nutritional status, physical activity and disease cycle, and the change of plasma FFA level with the development of diabetes is not clear, it may not be able to accurately reflect the change of FFAs in blood by one measurement. Finally, we did not perform follow-up and correlation analysis of final events in our study. Therefore, further study with large sample size may be needed to confirm our findings.

## Conclusions

In conclusion, data from our study verified that elevated FFA levels appeared associated with both the presence and severity of CAD and CAP in patients with T2DM, suggesting that plasma FFA levels may be a useful biomarker for improving management of patients with T2DM.

## Data Availability

The datasets used and/or analysed during the current study are not publicly available but are available from the corresponding author on reasonable request.

## Abbreviations

FFAs: Free fatty acids
T2DM: Type 2 diabetes mellitus
CAD: Coronary artery disease
AS: Atherosclerosis
ASCVD: Atherosclerotic cardiovascular disease
CAP: Carotid atherosclerotic plaque
BMI: Body mass index
HTN: Hypertension
ACEI: Angiotensin converting enzyme inhibitors
ARB: Angiotensin receptor blocker
FPG: Fasting plasma glucose
TC: Total cholesterol
TG: Triglyceride
HDL-C: high-density lipoprotein cholesterol
LDL-C: low-density lipoprotein cholesterol
apoA: Apolipoprotein A
apoB: Apolipoprotein B
HbA1c: Hemoglobin A1C
IMT: Intima-media thickness
LVEF: Left ventricular ejection fraction
